# Supplementary material for: In Silico Molecular Docking and Dynamics Simulation Analysis of Potential Histone Lysine Methyl Transferase Inhibitors for Managing β-Thalassemia
Source: Molecules. 2023 Oct 25;28(21):7266. doi: 10.3390/molecules28217266 (PMC10650625; doi:10.3390/molecules28217266)
Supplement: Supplementary file 1 [file molecules-28-07266-s001.zip › molecules-2642812-supplementary.pdf]

Supplementary Information

Table S1. The table presents the results of various interactions of F80, N47, TP1, and TP2 with EHMT2 attained during docking and MDS studies.

Ligand name	Hydrogen and hydrophobic interaction	Distance (Å)	Category	Type
**F80**	A:CYS1098:SG - C:F801506:N3	3.55644	Hydrogen bond	Conventional Hydrogen Bond
	C:F801506:N4 - A:LEU1086:O	2.92271	Hydrogen bond	Conventional Hydrogen Bond
	C:F801506:C10 - A:ASP1083:OD2	3.26131	Hydrogen bond	Carbon Hydrogen Bond
	C:F801506:C10 - A:PHE1158:O	3.65828	Hydrogen bond	Carbon Hydrogen Bond
	C:F801506:C16 - A:SER1084:O	3.4725	Hydrogen bond	Carbon Hydrogen Bond
	C:F801506:C4 - A:ASP1088:OD1	3.52444	Hydrogen bond	Carbon Hydrogen Bond
	A:ARG1157:NH1 - C:F801506	3.13087	Electrostatic	Pi-Cation
	A:LEU1086:CD1 - C:F801506	3.98444	Hydrophobic	Pi-Sigma
	A:ALA1077 - C:F801506:C2	4.15024	Hydrophobic	Alkyl
	C:F801506:C10 - A:ILE1161	4.06518	Hydrophobic	Alkyl
	C:F801506:C10 - A:LYS1162	4.07173	Hydrophobic	Alkyl
	C:F801506:C4 - A:VAL1096	4.44062	Hydrophobic	Alkyl
	C:F801506:C4 - A:CYS1098	5.16483	Hydrophobic	Alkyl
	A:PHE1158 - C:F801506:C10	4.44102	Hydrophobic	Pi-Alkyl
	A:PHE1158 - C:F801506	5.01756	Hydrophobic	Pi-Alkyl
	C:F801506 - A:ALA1077	4.0023	Hydrophobic	Pi-Alkyl
	C:F801506 - A:CYS1098	5.05205	Hydrophobic	Pi-Alkyl
	C:F801506 - A:LEU1086	4.84546	Hydrophobic	Pi-Alkyl
	C:F801506 - A:ARG1157	4.78416	Hydrophobic	Pi-Alkyl
**N47**	C:N471506:C6 - A:ASP1088:OD1	3.50018	Hydrogen bond	Carbon hydrogen bond
	C:N471506:C12 - A:ASP1088:OD1	3.36383	Hydrogen bond	Carbon hydrogen bond
	C:N471506:C - A:TYR1154	3.73454	Hydrophobic	Pi-Sigma
	C:N471506:C18 - A:PHE1087	3.74022	Hydrophobic	Pi-Sigma

	A:PHE1087 - C:N471506	5.11678	Hydrophobic	Pi-Pi T-shaped
	A:ALA1077 - C:N471506	3.76422	Hydrophobic	Alkyl
	A:VAL1096 - C:N471506	4.73503	Hydrophobic	Alkyl
	A:CYS1098 - C:N471506	4.481	Hydrophobic	Alkyl
	C:N471506 - A:LEU1086	4.88529	Hydrophobic	Alkyl
	C:N471506 - A:LEU1086	4.85558	Hydrophobic	Pi-Alkyl
	C:N471506 - A:ARG1157	4.66909	Hydrophobic	Pi-Alkyl
**TP1**	C:SUB1:H31 - A:ASP1083:OD2	2.57287	Hydrogen bond	Conventional hydrogen bond
	A:ARG1157:NH1 - C:SUB1	3.39363	Electrostatic	Pi-Cation
	A:ALA1077:CB - C:SUB1	3.68893	Hydrophobic	Pi-Sigma
	A:LEU1086:CD1 - C:SUB1	3.8528	Hydrophobic	Pi-Sigma
	A:PHE1158:CB - C:SUB1	3.90749	Hydrophobic	Pi-Sigma
	C:SUB1 - A:ARG1157	5.13898	Hydrophobic	Pi-Alkyl
	C:SUB1 - A:ILE1161	4.80605	Hydrophobic	Pi-Alkyl
	C:SUB1 - A:LEU1086	5.49342	Hydrophobic	Pi-Alkyl
	C:SUB1 - A:CYS1098	4.84415	Hydrophobic	Pi-Alkyl
	C:SUB1 - A:ARG1157	5.16907	Hydrophobic	Pi-Alkyl
**TP2**	A:ARG1157:NE - C:SUB1:O1	3.28514	Hydrogen bond	Conventional hydrogen bond
	C:SUB1:H38 - A:ASP1083:O	1.82219	Hydrogen bond	Conventional hydrogen bond
	C:SUB1:H49 - A:SER1084:O	2.29565	Hydrogen bond	Conventional hydrogen bond
	C:SUB1:C26 - A:ASP1088:OD1	3.45164	Hydrogen bond	Carbon hydrogen bond
	A:ARG1157:NH1 - C:SUB1	3.26174	Electrostatic	Pi-Cation
	C:SUB1:N7 - A:TYR1154	3.90554	Electrostatic	Pi-Cation
	A:ASP1083:OD2 - C:SUB1	3.70387	Electrostatic	Pi-Anion
	A:ALA1077:CB - C:SUB1	3.79309	Hydrophobic	Pi-Sigma
	A:LEU1086:O - C:SUB1	2.96418	Other	Pi-Lone pair

A:TYR1154 - C:SUB1	4.95762	Hydrophobic	Pi-Pi stacked
A:PHE1158 - C:SUB1	5.04117	Hydrophobic	Pi-Pi T-shaped
A:ALA1077 - C:SUB1	4.91676	Hydrophobic	Alkyl
A:LEU1086 - C:SUB1	4.65418	Hydrophobic	Alkyl
C:SUB1:C26 - A:VAL1096	4.07355	Hydrophobic	Alkyl
C:SUB1:C26 - A:CYS1098	4.33742	Hydrophobic	Alkyl
C:SUB1:C23 - A:ILE1161	3.88775	Hydrophobic	Alkyl
C:SUB1 - A:LEU1086	5.47462	Hydrophobic	Pi-Alkyl
C:SUB1 - A:ARG1157	5.0361	Hydrophobic	Pi-Alkyl
C:SUB1 - A:ILE1161	5.00598	Hydrophobic	Pi-Alkyl
C:SUB1 - A:CYS1098	4.9569	Hydrophobic	Pi-Alkyl

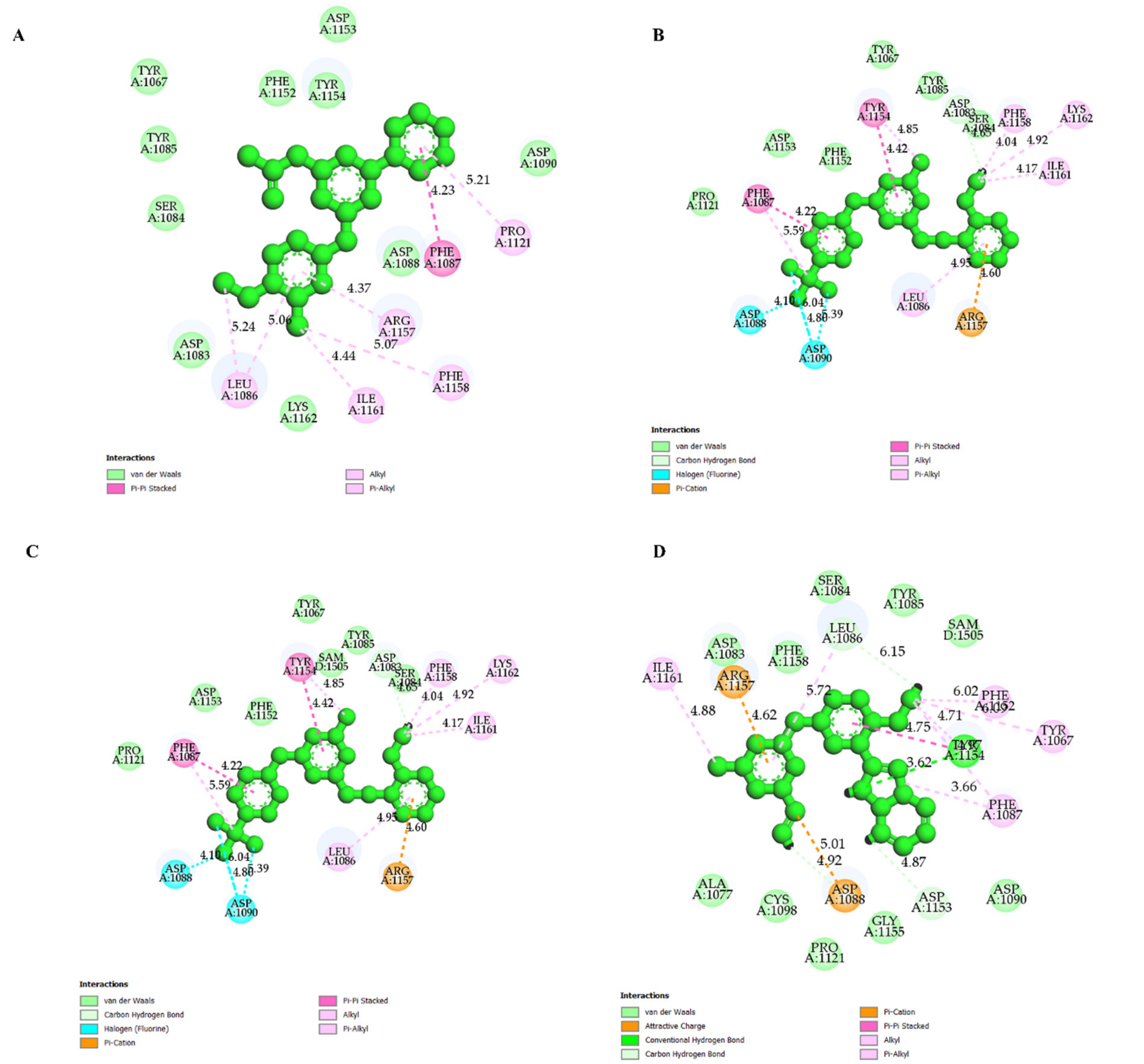

Figure S1: Molecular docking interaction analysis of selected compounds with EHMT2. Green color ball and stick representation denoted different ligands. The various interaction formed in each diagram is represented in different colors and is denoted as a footnote below. **A**. Tp3 docked with EHMT2. **B**. Tp4 docked with EHMT2. **C**. Tp5 docked with EHMT2. **D**. Tp6 docked with EHMT2.

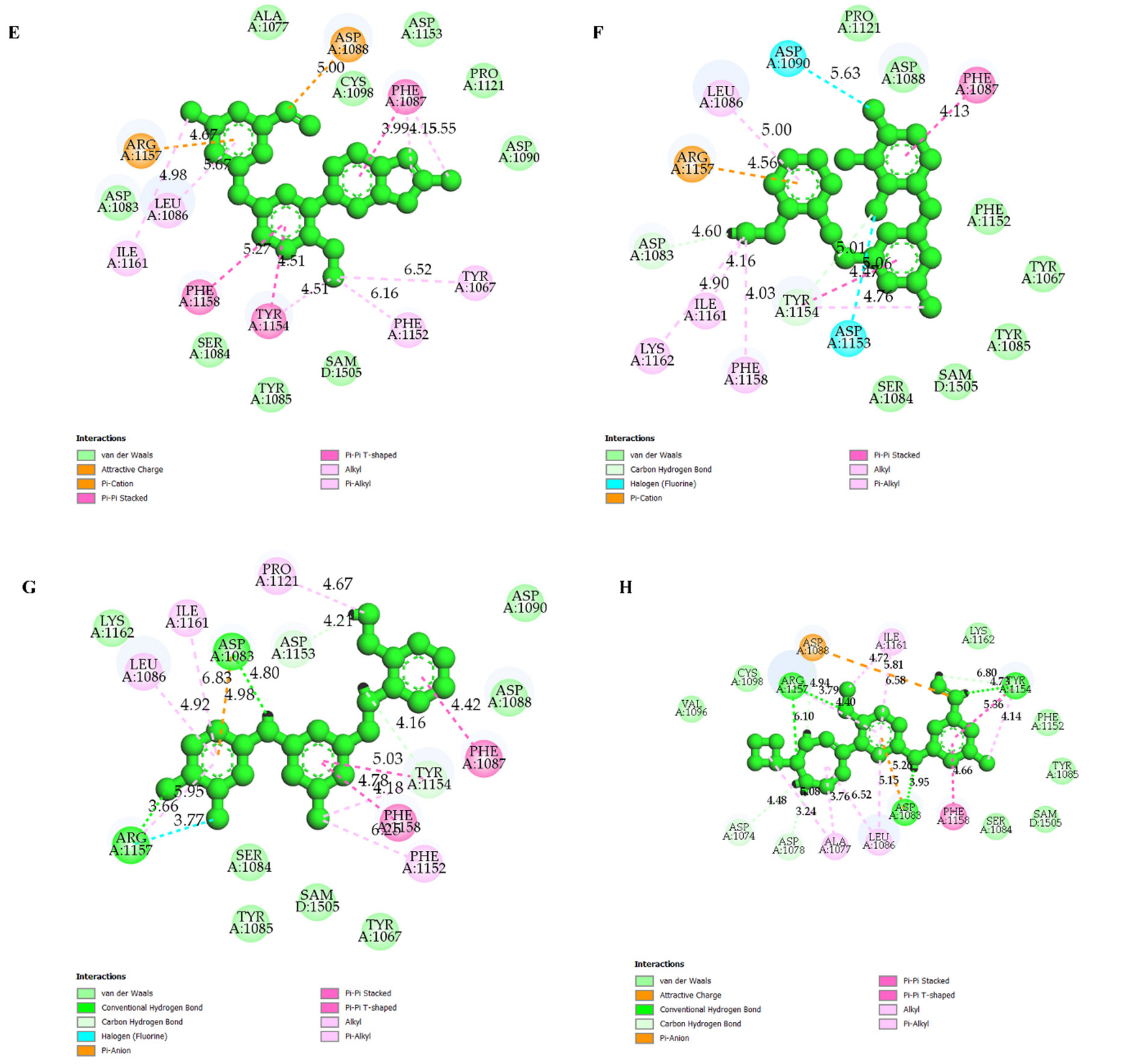

Figure S2: Molecular docking interaction analysis of selected compounds with EHMT2. Green color ball and stick representation denoted different ligands. The various interaction formed in each diagram is represented in different colors and is denoted as a footnote below. **E**. Tp7 docked with EHMT2. **F**. Tp8 docked with EHMT2. **G**. Tp9 docked with EHMT2. **H**. Tp10 docked with EHMT2.

Table S2. The table presents the results of various pharmacokinetic properties of the selected drug compounds obtained using ADMET2.0

F80

Adsorption		Metabolism		Distribution		Excretion	
**Properties**	**Value**	**Properties**	**Value**	**Properties**	**Value**	**Properties**	**Value**
Caco2 permeability	-4.748	CYP1A2 inhibitor	-	PPB	39.051%	CL	5.313
MDCK permeability	3.8	CYP1A2 substrate	++	VD	2.061	T1/2	0.652
Pgp-inhibitor	+++	CYP2C19 inhibitor	--	BBB penetration	-		
Pgp-substrate	+++	CYP2C19 substrate	---	Fu	74.862%		
HIA	---	CYP2C9 inhibitor	---				
		CYP2C9 substrate	---				
		CYP2D6 inhibitor	---				
		CYP2D6 substrate	---				
		CYP3A4 inhibitor	--				
		CYP3A4 substrate	++				

N47

Adsorption		Metabolism		Distribution		Excretion	
**Properties**	**Value**	**Properties**	**Value**	**Properties**	**Value**	**Properties**	**Value**
Caco2 permeability	-4.761	CYP1A2 inhibitor	--	PPB	59.647%	CL	5.496
MDCK permeability	6.8	CYP1A2 substrate	+++	VD	1.720	T1/2	0.521
Pgp-inhibitor	---	CYP2C19 inhibitor	---	BBB penetration	++		
Pgp-substrate	+++	CYP2C19 substrate	---	Fu	51.753%		

HIA	---	CYP2C9 inhibitor	---				
		CYP2C9 substrate	---				
		CYP2D6 inhibitor	---				
		CYP2D6 substrate	--				
		CYP3A4 inhibitor	---				
		CYP3A4 substrate	+++				

TP1

Adsorption		Metabolism		Distribution		Excretion	
**Properties**	**Value**	**Properties**	**Value**	**Properties**	**Value**	**Properties**	**Value**
Caco2 permeability	-4.763	CYP1A2 inhibitor	+++	PPB	99.794%	CL	5.193
MDCK permeability	2.4	CYP1A2 substrate	++	VD	2.065	T1/2	0.174
Pgp-inhibitor	++	CYP2C19 inhibitor	++	BBB penetration	--		
Pgp-substrate	---	CYP2C19 substrate	---	Fu	0.927%		
HIA	---	CYP2C9 inhibitor	+				
		CYP2C9 substrate	---				
		CYP2D6 inhibitor	++				
		CYP2D6 substrate	--				
		CYP3A4 inhibitor	+				
		CYP3A4 substrate	+				

TP2

Adsorption		Metabolism		Distribution		Excretion	
**Properties**	**Value**	**Properties**	**Value**	**Properties**	**Value**	**Properties**	**Value**
Caco2 permeability	-4.715	CYP1A2 inhibitor	++	PPB	93.952%	CL	4.507
MDCK permeability	5.7	CYP1A2 substrate	+++	VD	0.989	T1/2	0.457
Pgp-inhibitor	+++	CYP2C19 inhibitor	+	BBB penetration	--		
Pgp-substrate	+++	CYP2C19 substrate	---	Fu	6.730%		
HIA	-	CYP2C9 inhibitor	--				
		CYP2C9 substrate	---				
		CYP2D6 inhibitor	---				
		CYP2D6 substrate	--				
		CYP3A4 inhibitor	+				
		CYP3A4 substrate	+++				

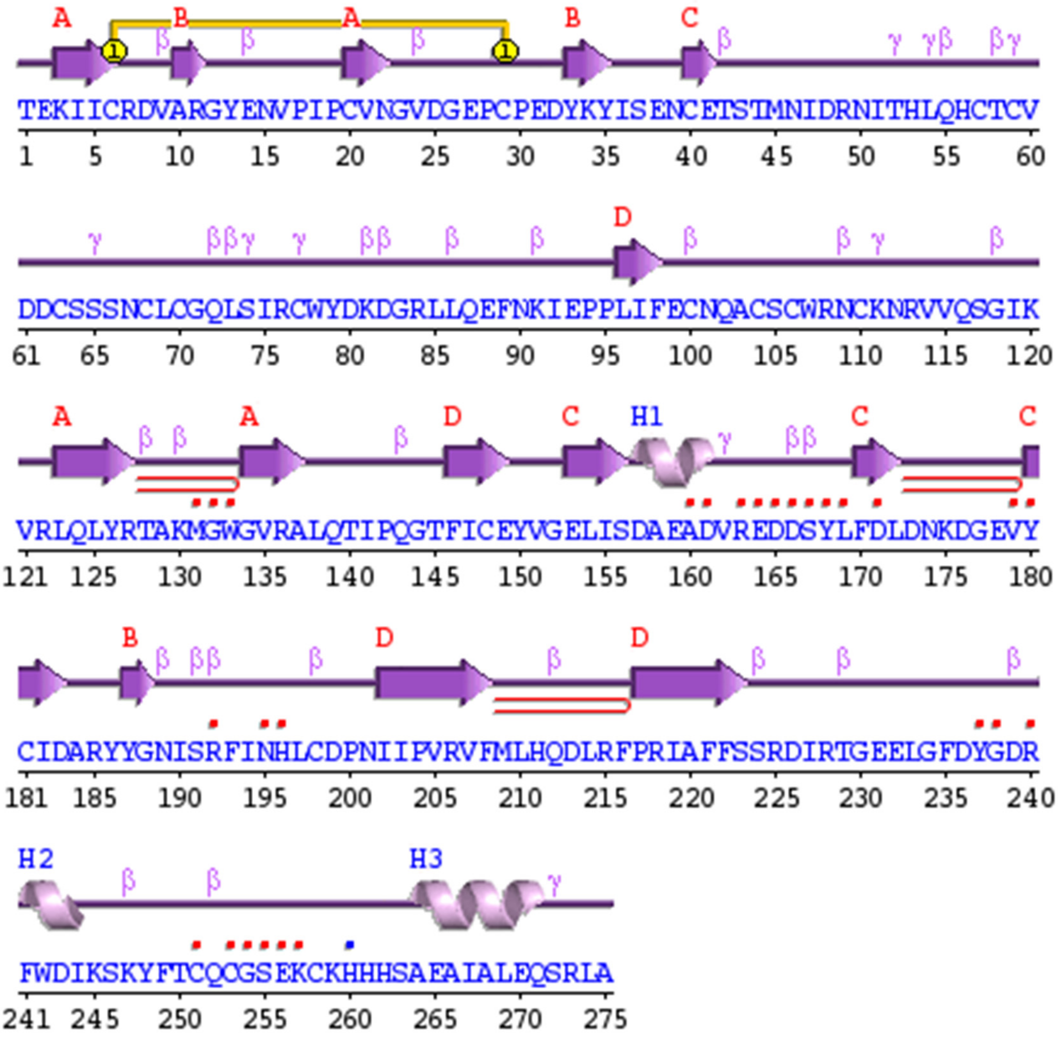

Figure S3: Secondary structure representation of EHMT2 protein. The figure shows the presence of β-sheets, helixes corresponding to the amino acids. The red asterisk denotes the residues catalytically essential and interacts with ligands.

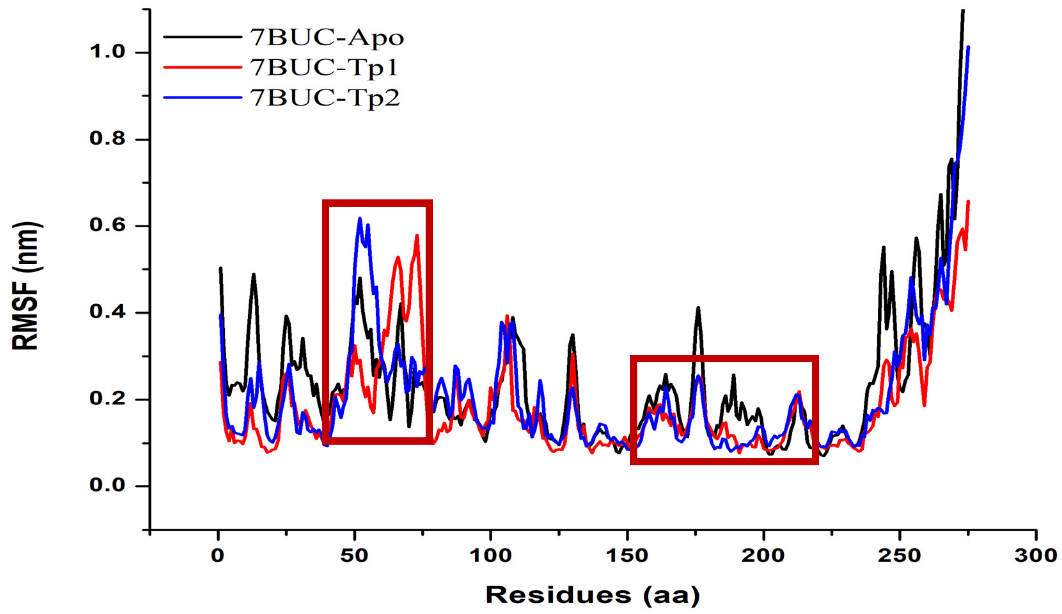

Figure S4: RMSF plot of docked complexes generated through MDS at 200 ns. The black color plot indicates the EHMT2 apo form. The red color plot indicates the Tp1 and the Blue color indicates the Tp2.

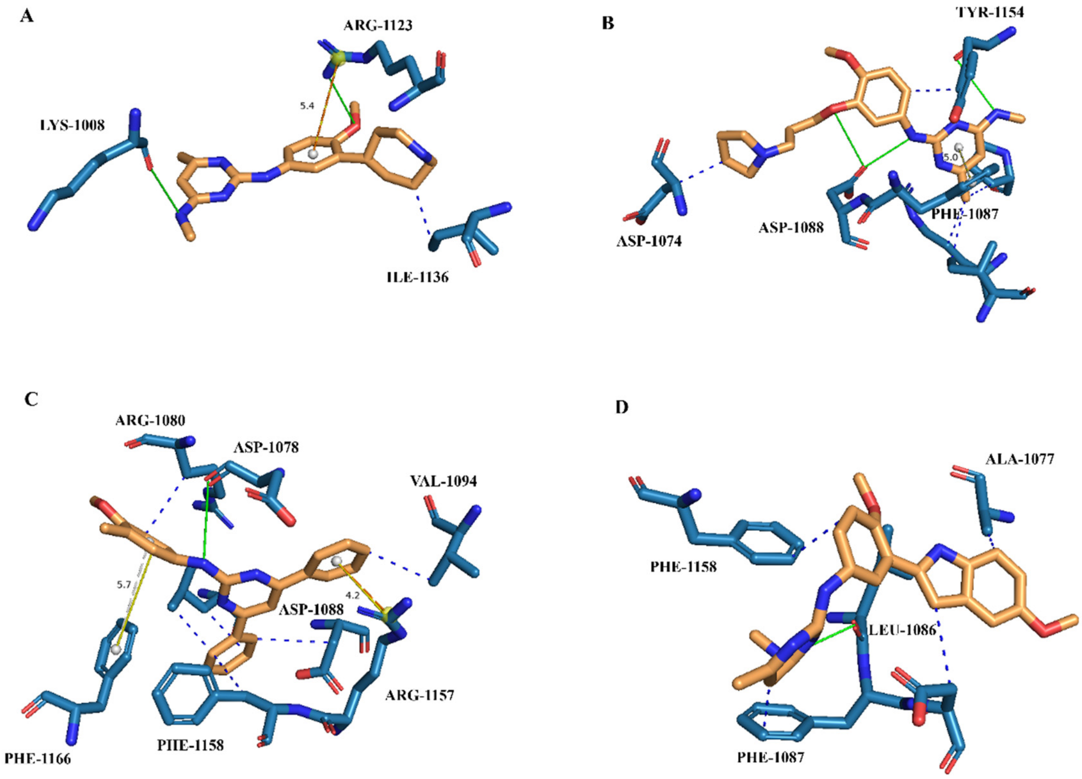

Figure S5. Molecular interactions analysis of docked complexes generated through MDS at 200 ns. (A). Hydrogen, hydrophobic bonds formed between 7BUC complexed with F80. (B) Hydrogen, π-π stacking, and hydrophobic bonds formed between 7BUC complexed with N47. (C) Hydrogen, cation- π, π-π stacking and hydrophobic bonds formed between 7BUC complexed with TP1, and (D) Hydrogen, hydrophobic bonds formed between 7BUC complexed with TP2.

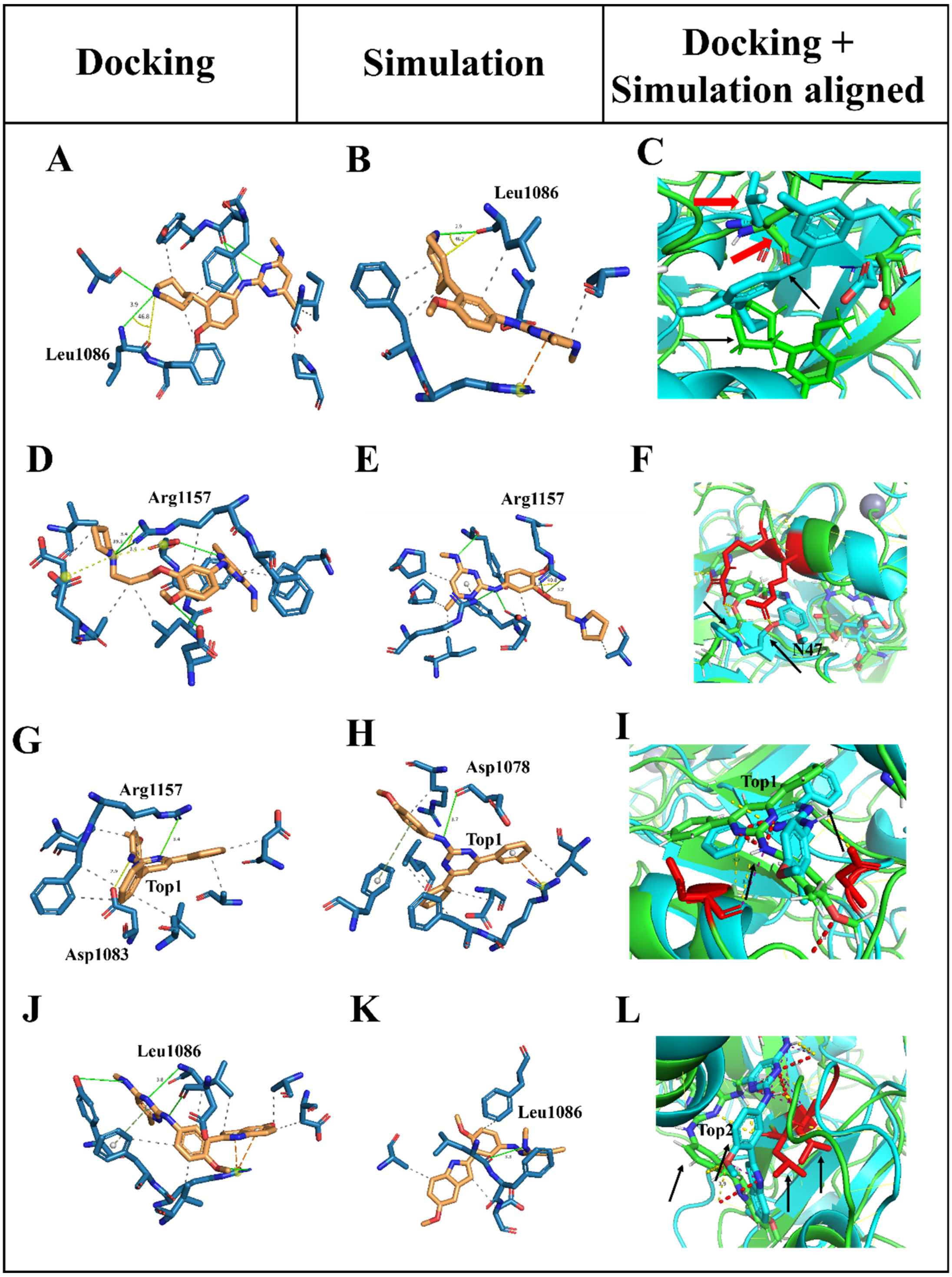

Figure S6 Comparative molecular interactions analysis of docked complexes generated through MDS at 200 ns. (A). Interactions formed between 7BUC complexed with F80 while docking. (B) Interactions formed between 7BUC complexed with F80 while simulation (C) Superimposition of docked and simulated F80 with 7BUC. (D). Interactions formed between 7BUC complexed with N47 while docking. (E) Interactions formed between 7BUC complexed with N47 while simulation (F) Superimposition of docked and simulated N47 with 7BUC. (G). Interactions formed between 7BUC complexed with Tp1 while docking. (H) Interactions formed between 7BUC complexed with Tp1 while simulation (I) Superimposition of docked and simulated Tp1 with 7BUC. (J). Interactions formed between 7BUC complexed with Tp2 while docking. (K) Interactions formed between 7BUC complexed with Tp2 while simulation (L) Superimposition of docked and simulated Tp2 with 7BUC.
